# *Lrig1* expression identifies quiescent stem cells in the ventricular-subventricular zone from postnatal development to adulthood and limits their persistent hyperproliferation

**DOI:** 10.1186/s13064-022-00169-1

**Published:** 2023-01-11

**Authors:** Hyung-song Nam, Mario R. Capecchi

**Affiliations:** Department of Human Genetics, University of Utah School of Medicine, Salt Lake City, UT 84112-5331 USA

**Keywords:** Adult neurogenesis, C57BL/6J, Gene knock-out, Genetic inducible fate mapping, Inbred mice, *Lrig1*, Neural development, Quiescence, Stem cells, Ventricular-subventricular zone

## Abstract

**Background:**

We previously identified *Leucine-rich repeats and immunoglobulin-like domains 1* (*Lrig1*) as a marker of long-term neurogenic stem cells in the lateral wall of the adult mouse brain. The morphology of the stem cells thus identified differed from the canonical B1 type stem cells, raising a question about their cellular origin. Thus, we investigated the development of these stem cells in the postnatal and juvenile brain. Furthermore, because *Lrig1* is a known regulator of quiescence, we also investigated the effect(s) of its deletion on the cellular proliferation in the lateral wall.

**Methods:**

To observe the development of the *Lrig1*-lineage stem cells, genetic inducible fate mapping studies in combination with thymidine analog administration were conducted using a previously published *Lrig1*^*T2A-iCreERT2*^ mouse line. To identify the long-term consequence(s) of *Lrig1* germline deletion, old *Lrig1* knock-out mice were generated using two different *Lrig1* null alleles in the C57BL/6J background. The lateral walls from these mice were analyzed using an optimized whole mount immunofluorescence protocol and confocal microscopy.

**Results:**

We observed the *Lrig1*-lineage labeled cells with morphologies consistent with neurogenic stem cell identity in postnatal, juvenile, and adult mouse brains. Interestingly, when induced at postnatal or juvenile ages, morphologically distinct cells were revealed, including cells with the canonical B1 type stem cell morphology. Almost all of the presumptive stem cells labeled were non-proliferative at these ages. In the old *Lrig1* germline knock-out mice, increased proliferation was observed compared to wildtype littermates without concomitant increase in apoptosis.

**Conclusions:**

Once set aside during embryogenesis, the *Lrig1*-lineage stem cells remain largely quiescent during postnatal and juvenile development until activation in adult age. The absence of premature proliferative exhaustion in the *Lrig1* knock-out stem cell niche during aging is likely due to a complex cascade of effects on the adult stem cell pool. Thus, we suggest that the adult stem cell pool size may be genetically constrained via *Lrig1*.

**Supplementary Information:**

The online version contains supplementary material available at 10.1186/s13064-022-00169-1.

## Background

In the laboratory model organism *Mus musculus*, neural progenitor cells generate the various and multitude cell types that form the cellular architecture of the mammalian brain during embryonic corticogenesis (recently reviewed in [1]). At more than half-way through this process, some of the embryonic neural progenitor cells are set aside to later become adult neural stem cells [2–4]. Then, in the adult brain, these adult neural stem cells continue to generate neurons in a process termed adult neurogenesis (reviewed in [5]).

The adult neural stem cells are observed in at least two major niches, in the lateral wall lining the lateral ventricles and in the hippocampal dentate gyrus. We studied the adult stem cells in the lateral wall for the following reasons. First, in terms of the numbers of adult newborn neurons generated, the lateral wall is the largest germinal niche in the adult brain (reviewed in [6]). Second, for mice, olfaction is a sensory modality perhaps more important than vision (reviewed in [7]), and the vast numbers of neurons generated from the lateral wall [8, 9] are likely involved in olfaction because they form circuits in the olfactory bulb (reviewed in [10]). Third, the proliferating cells in the ventricular wall, whether they are stem or more differentiated precursor cells, are more susceptible to generate brain tumors when transformed [11].

The lateral wall adult neural stem cells are located in the ventricular-subventricular zone (reviewed in [12]). Sustained progress in the field means that the adult neural stem cells have now been defined molecularly with single cell RNA sequencing [13–18]. Nevertheless, the relationship between the single cell transcriptomes and the neuroanatomical characteristics of the stem cells remains unclear. For example, pioneering studies have identified two seemingly different stem cell populations, the branched B type stem cell [19] and the ventricle-contacting B1 type stem cell [20], and yet, the lineage relationship between these cells and whether these cells differ molecularly are not known.

In our line of research, we followed the threads of our previous work that identified a genetic marker for a neurogenic stem cell type resembling the first stem cell type mentioned above, the branched B type stem cell [21]. These *Lrig1*-lineage stem cells generated neuroblasts and neurons as late as 2 years and 6 months of age [21–23]. Here, we have performed additional lineage tracing studies using our mouse model, the *Lrig1*^*T2A-iCreERT*2^ mouse, and discovered that the cells resembling the second stem cell type, the B1 type stem cell, can also be revealed. This meant that we could trace the origin of these two morphotypes, and we found that the two subsets originate from a single progenitor pool, the postnatal radial glial cells [24]. In addition, we determined the long-term effect(s) of knocking out the marker gene, *Lrig1*, and suggest that it constrains the adult neural stem cell pool size.

## Methods

### Mouse experimentation

Mice were housed in Thoren rack cages. After weaning at 3 weeks of age, 3–4 same-sex littermates were housed in each cage with bedding and paper nestlets as environmental enrichment. Mice were transferred to fresh cages every 2 weeks. Food and water were provided ad libitum. The food was a custom formulation referred to as the Capecchi diet (Envigo Teklad # 3980X).

Mouse lines were generated in-house or obtained from outside sources (see Results section for literature references, see next section for repository stock #). All mouse lines were backcrossed for at least 4 generations to C57BL/6J mice. The C57BL/6J mouse stock was obtained from The Jackson Laboratory (strain # 000664). New mice were regularly purchased to refresh the stock.

The mice in the experimental cohorts were not systematically randomized because the mice were bred to be genetically as similar as practically possible to each other. Both sexes were included in the cohorts.

### Mouse genotyping

Ear clips from the mice were processed by HotSHOT method [25], then genotyped by PCR with Taq (Takara, Azura Genomics). The standardized PCR cycling program was 95 °C 3 min, (95 °C 20 sec, 60 °C 30 sec, 72 °C 1 min) × 30 cycles, 72 °C 2 min. Primer sequences follow.

Tg(Nr2e1-EGFP) (MMRRC stock # 033024-UCD)

5′-AAGGGCATCGACTTCAAGGA-3′

5′-CTTGTACAGCTCGTCCATGC-3′

339 bp EGFP

Capecchi *Lrig1*^*T2A-iCreERT2*^ (MMRRC stock # 069516-UCD)

5′-AGCTCATGGAAGACGCCATA-3′

5′-CCAGATGCCACTCCTCTAGC-3′

5′-CCGGATCCATTATGTACCTGAC-3′

302 bp wildtype allele, 211 bp knock-in allele

Coffey *Lrig1*^*creERT2*^ (Jax stock # 018418)

5′-GACTCGCTGGACTGCAGT-3′

5′-CCGTCTCACATGCACACAAA-3′

5′-CGAGTGATGAGGTTCGCAAG-3′

5′-TTCACCGGCATCAACGTTTT-3′

483 bp wildtype allele, 332 bp cre

*Pdgfrb*^*P2A-creERT2*^ (Jax stock # 030201)

5′-TTTCCCCGGTTCCTTTCTGA-3′

5′-TTGAGTGCTGCAAACCAAGG-3′

5′-ATCTTCAGGTTCTGCGGGAA-3′

349 bp wildtype allele, ~450 bp knock-in allele

*Rosa26*^*Ai14*^ (Allen Institute for Brain Science)

5′-GCACTTGCTCTCCCAAAGTC-3′

5′-GGCGGATCACAAGCAATAAT-3′

5′-TTATGTAACGCGGAACTCCA-3′

445 bp wildtype allele, 320 bp knock-in allele

EUCOMM *Lrig1*^*Δ*^ (EMMA stock # EM:05375, FLP then cre recombined)

5′-AACACAGGCAAGAGGAAGGT-3′

5′-AGACCTGCTCTTCCTGTGTT-3′

1096 wildtype allele, 413 bp Δ allele

### Tamoxifen induction

Tamoxifen (Sigma) was solubilized in 90% peanut oil (Sigma) and 10% ethanol vehicle. Fresh tamoxifen formulation was prepared about an hour before injection by warming the suspension at 37 °C and solubilizing with a Branson sonifier and vortexer (see below for the stock concentrations). Thorough sonification was critically necessary for efficient induction. For mice older than postnatal day 21, every mouse in a cohort was weighed, then intraperitoneally injected once with a calculated volume of the tamoxifen formulation. Inductions were most reproducible at volumes of 50–100 μl. Thus, we injected similar low volumes while varying the tamoxifen stock concentration. Inductions of male and female mice were comparable because the injection volumes were adjusted for the mouse weight differences. All mice in the cohort were injected in one session and returned to fresh cages. The cages were changed again at 3 days after the injection. For mice older than postnatal day 0, pups were injected subcutaneously ~20 μl of the tamoxifen formulation late in the day. The cages were changed at 3 days after the injection. These inductions were more variable.

Tamoxifen stock concentrations follow. Juvenile to adult inductions: Capecchi *Lrig1* reporter 170 mg/kg, 54.8 mg/ml; Coffey *Lrig1* reporter 120 mg/kg, 43.5 mg/ml; *Pdgfrb* reporter 13.5 mg/kg, 3.8 mg/ml; Capecchi *Lrig1* reporter 214 mg/kg, 60 mg/ml; Capecchi *Lrig1* reporter 60 mg/kg, 8.6 mg/ml. Postnatal induction: Capecchi *Lrig1* reporter 20 mg/kg, 1 mg/ml.

### Thymidine analog administration

EdU (ethynyl deoxyuridine, Carbosynth) was administered in drinking water for 1 week at 0.15 mg/ml with 1% glucose to avoid taste aversion. In preliminary experiments, the EdU dose was titrated. High EdU dose, 0.8 mg/ml, reduced the number of ASCL1+, KI-67+, or DCX+ cells in the lateral wall as determined by whole mount immunofluorescence analyses, but the low dose, 0.15 mg/ml, did not and was minimally toxic [26]. BrdU (bromo deoxyuridine, Carbosynth) was administered in drinking water for 1 week at 0.8 mg/ml with 1% glucose.

### Whole mount immunofluorescence

Isofluorane-anesthetized mice were transcardially perfused with room-temperature PBS with 20 U/ml heparin then ice-cold 2% PLP fixative [27] composed of 2% formaldehyde, 75 mM lysine, 10 mM NaIO_4_, and 0.1 M phosphate buffer pH 7.4. Perfused brains were dissected out, rinsed in PBS, then bisected and further dissected in PBS to reveal the lateral wall [28]. The dissected brains were post-fixed overnight in 2% PLP at 4 °C on a nutator. The fixed brains were rinsed in PBS, then blocked with 0.3 M glycine in PBS pH 7.4 overnight at 4 °C on nutator. The brains could be stored refrigerated at this point. The brains were trimmed in PBS, and then permeabilized with 0.5% Triton X-100 in PBS at room temperature. When using goat secondaries, the brains were blocked with 10% normal goat serum (Vector Labs), 20 μg/ml goat anti-mouse IgG F(ab) fragment (Jackson ImmunoResearch), 0.5% BSA (Jackson ImmunoResearch), and 0.1% TX-100 in PBS. When using donkey secondaries, the brains were blocked with 10% normal donkey serum (Jackson ImmunoResearch), 20 μg/ml donkey anti-mouse IgG F(ab) fragment (Jackson ImmunoResearch), 25% CytoQ (Innovex Biosciences), and 0.1% TX-100 in PBS. The block was performed at 4 °C on nutator. After brief washes in PBS + 0.1% TX-100 (PBST) at room temperature, the brains were nutated at 4 °C for 48 h with primary antibodies. When using goat secondaries, the antibodies were diluted in 1% normal goat serum, 0.5% BSA, and 0.1% TX-100 in PBS. When using donkey secondaries, the antibodies were diluted in 1% normal donkey serum, 25% CytoQ, and 0.1% TX-100 in PBS. After four washes in PBST at room temperature, the brains were again nutated with cross-adsorbed DyLight 405, Alexa 488, Rhodamine Red-X, and/or Alexa 647 secondary antibodies (Jackson ImmunoResearch, Invitrogen, see below) as above for 24 h. The brains were washed 3 times in PBST then 1 time in TBST (100 mM Tris pH 8.5, 150 mM NaCl, 0.1% TX-100) at room temperature. A slice was cut with a custom 3D printed jig and a razor or with a scalpel freehand. The slice was trimmed in TBS (minus TX-100) then coverslipped with a #1.5 cover glass (Fisher Scientific) within a 0.5 mm spacer (Invitrogen) in a mounting media composed of 10% w/v Mowiol (Polysciences), 25% w/v glycerol (Sigma), and 100 mM Tris pH 8.5.

### Thymidine analog detection on whole mounts

Click chemistry for EdU detection [29] was performed after permeabilization and before the antibody staining with Alexa 488-picolyl azide (Invitrogen) for 1 h at room temperature on nutator. To reduce the background from copper sulfate, the brains were washed with distilled water before the click chemistry and with TBST (see above for composition) after the click chemistry. For BrdU detection, the brains were pre-treated with 2 N hydrochloric acid for 40 min at 37 °C then with 0.1 M sodium borate pH 8.5 for 20 min at room temperature.

### Primary antibodies

ASCL1 (MASH1) mouse monoclonal clone 24B72D11.1 BD Biosciences 556604 1:500

β-CATENIN mouse monoclonal clone 14/Beta-catenin BD Biosciences 610153 1:500

BrdU mouse monoclonal clone MoBU-1 BioLegend 317902 1:500

Cleaved CASP3 rabbit polyclonal Cell Signaling Technology 9661 T 1:200

DCX guinea pig polyclonal Milipore AB2253 1:5000

GFAP chicken polyclonal BioLegend (Covance) 829401 1:500

GFP chicken polyclonal Rockland Immunochemicals 600–901-215 1:500

KI-67 rat monoclonal clone SolA15 Invitrogen 14–5698-82 1:500

RFP goat polyclonal Rockland Immunochemicals 200–101-379 1:500

RFP guinea pig polyclonal Frontier Institute MSFR105900 1:500

RFP rabbit polyclonal Rockland Immunochemicals 600–401-379 1:500

S100 rabbit polyclonal Invitrogen PA5–16257 (product discontinued) 1:500

VCAM1 rat monoclonal clone 429 (MVCAM.A) BD Biosciences 553330 1:200

### Secondary antibodies

DyLight™ 405 Goat Anti-Chicken IgY (IgG) (H + L) Jackson ImmunoResearch 103–475-155 1:250

DyLight™ 405 Goat Anti-Rat IgG (H + L) Jackson ImmunoResearch 112–475-167 1:250

Alexa Fluor® 488 Donkey Anti-Rabbit IgG (H + L) Jackson ImmunoResearch 711–545-152 1:500

Alexa Fluor® 488 Goat Anti-Chicken IgY (IgG) (H + L) Jackson ImmunoResearch 103–545-155 1:500

Alexa Fluor™ 488 Goat anti-Guinea Pig IgG (H + L) Invitrogen A-11073 1:500

Alexa Fluor® 488 Goat Anti-Rat IgG (H + L) Jackson ImmunoResearch 112–545-167 1:500

Rhodamine Red™-X Donkey Anti-Goat IgG (H + L) Jackson ImmunoResearch 705–295-147 1:250

Rhodamine Red™-X Goat Anti-Rabbit IgG (H + L) Jackson ImmunoResearch 111–295-144 1:500

Rhodamine Red™-X Goat Anti-Rat IgG (H + L) Jackson ImmunoResearch 112–295-167 1:500

Alexa Fluor™ 555 Goat anti-Guinea Pig IgG (H + L) Invitrogen A-21435 1:500

Alexa Fluor™ 647 Donkey anti-Rabbit IgG (H + L) Invitrogen A-31573 1:500

Alexa Fluor® 647 Goat Anti-Mouse IgG (H + L) Jackson ImmunoResearch 115–605-166 1:500

### Confocal imaging

Images were acquired with a Leica TCS SP5 confocal microscope and processed with Fiji [30]. The microscope was equipped with the following lasers, 405 diode, argon, HeNe 543, HeNe 594, and HeNe 633, as well as four PMT’s, and a TD 488/543/633 dichroic. The objectives utilized were HC PL FLUOTAR 10× 0.3 dry, HCX PL APO CS 20× 0.7 dry UV, HCX PL APO CS 40× 1.25 oil UV, and HCX PL APO CS 63× 1.4 oil UV.

### Cell counting

Cells were counted using Fiji or Imaris (Oxford Instruments) as in our previous work [21].

### Statistics

Measured and counted values are represented as mean ± standard deviation. Statistical significance was calculated in R [31] with scripts using “shapiro.test,” “t.test,” “wilcox.test,” “aov,” “kruskal.test,” “pairwise.t.test,” “pairwise.wilcox.test,” and “ks.test” as appropriate. Briefly, normality was determined with Shapiro-Wilk test. For pairwise comparisons, Student’s t test or Mann-Whitney U test were performed depending on the normality of the samples. For multiple comparisons, differences in the samples were determined with ANOVA or Kruskal-Wallis test depending on the normality of the samples. Post-hoc tests were performed with pairwise Student’s t test or pairwise Mann-Whitney U test.

## Results

### In the lateral wall of adult mouse brain, *Lrig1* expression identifies quiescent stem cells that largely do not contact the ventricle

**Fig. 1:**
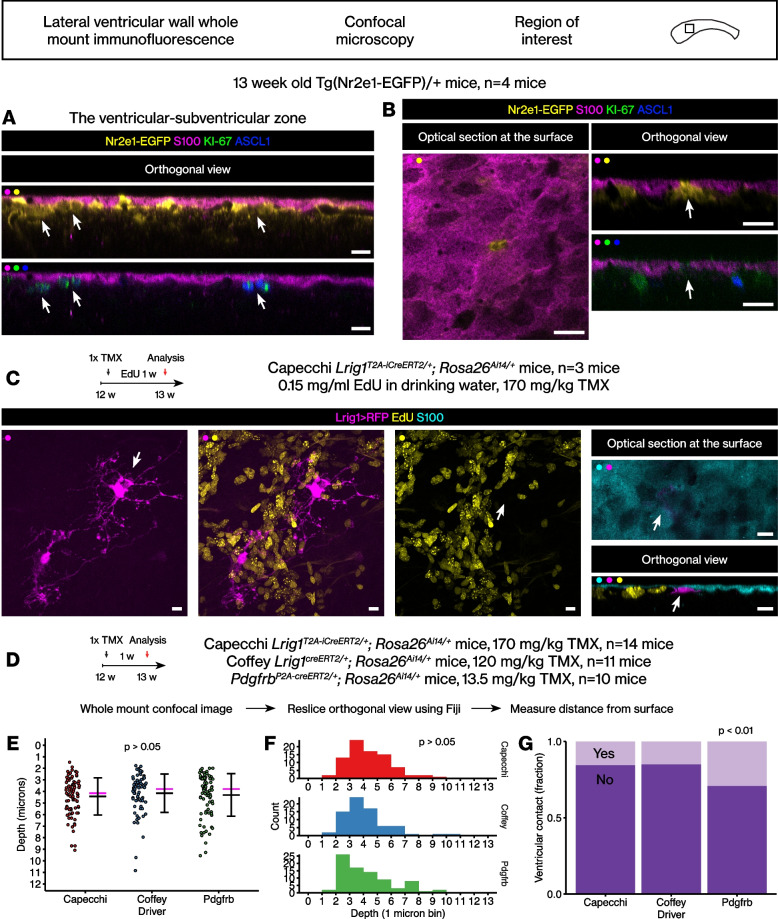
Quiescent *Lrig1*-lineage stem cells in the lateral wall of the adult mouse brain. **A** Control analysis of the Tg(Nr2e1-EGFP)/+ mouse brains. Orthogonal view after whole mount immunofluorescence and confocal microscopy. Scale bar, 10 μm. **B** An EGFP+ KI-67- ASCL1- cell filling the gap between S100+ ependymal cells, suggesting a B1 type stem cell identity. Scale bar, 10 μm. **C** The previously identified *Lrig1*-lineage stem cells largely did not incorporate EdU during one week pulse and did not contact the ventricle with an apical extension. Scale bar, 10 μm. **D** Analyses of additional mouse strains. **E** Dot plot of the distributions of the cell body locations. Black bars, mean ± standard deviation. Magenta bar, median. Student’s t test. **F** Histogram of the same distributions. Kolmogorov-Smirnov test. **G** Percentages of ventricle-contacting or non-ventricle-contacting cells. Chi-square test

To study the adult neural stem cells and their immediate progeny in the adult mouse brain, we analyzed the Tg(Nr2e1-EGFP) BAC transgenic mouse line from the GENSAT Project [32]. To our knowledge, this mouse line was not previously characterized in detail. In this mouse line, the regulatory sequences of a stem cell marker gene *Nr2e1* also known as *Tlx* [33] drive expression of EGFP marker protein in the neural stem cells and their immediate progeny. The hemizygous transgenic mice were analyzed at 13 weeks of age using an optimized whole mount immunofluorescence protocol and confocal imaging [21, 28]. To reveal the layer of ependymal cells and the gaps between them, we utilized an antibody against the S100 protein [20]. Consistent with previous work [34], this analysis revealed a subventricular or subependymal layer of EGFP+ cells, presumably neural stem cells and more differentiated precursor cells (Fig. 1A).

Immunoreactivities against the EGFP marker protein and proliferation markers such as KI-67 or ASCL1 were not mutually exclusive, with significant overlap (Fig. 1A). Most of the EGFP+ cells were KI-67+ and ASCL1+ (86 ± 2.5%, *n* = 4 ventricular walls from 4 mice), but the rest were KI-67- and ASCL1-. The cell densities were, all EGFP+ cells, 3905 ± 517 cells per mm^2^, *n* = 4 ventricular walls from 4 mice; EGFP+ KI-67+ ASCL1+ cells, 3373 ± 532 cells per mm^2^, *n* = 4 ventricular walls from 4 mice. The EGFP+ KI-67+ ASCL1+ triple-positive cells showed less intense EGFP immunoreactivity than the EGFP+ KI-67- ASCL1- cells. The observation of the EGFP+ KI-67- ASCL1- cells suggested the expression of *Nr2e1* can indeed be used to identify quiescent stem cells in the lateral ventricular wall, consistent with single cell RNA sequencing analyses [13, 16, 17].

As aforementioned, the S100 immunoreactivity revealed the gaps between the ependymal cells where the apical extensions of the B1 type stem cells are located. Consistent with the notion that at least some of the EGFP+ KI-67- ASCL1- cells are quiescent B1 type stem cells, we could easily identify the EGFP+ KI-67- ASCL1- cells filling the gaps between the S100+ ependymal cells (Fig. 1B). The cell bodies of the EGFP+ KI-67- ASCL1- B1 type stem cells were in the subventricular zone under the layer of the S100+ ependymal cells. Thus, we could identify the previously known population of the quiescent B1 type stem cells using the whole mount method [21, 28].

Next, we analyzed the brains from our *Lrig1*^*T2A-iCreERT2*^ mouse line [21] using the same method. The *Lrig1*^*T2A-iCreERT2/+*^*; Rosa26*^*Ai14/+*^ mice were induced once with tamoxifen at 12 weeks of age then perfused 1 week after. During that 1 week, the mice were also administered a thymidine analog, ethynyl deoxyuridine (EdU), in the drinking water at 0.15 mg/ml. This labeling paradigm labels the *Lrig1*-lineage cells in the young adult brain, and also reveals how many of them are in S-phase at the time of their labeling. First, confirming our previous observations in Nam and Capecchi, 2020, analysis of the lateral walls revealed only a few RFP-labeled B1 type stem cells (5.0 ± 1.7 cells per mm^2^, *n* = 3 ventricular walls from 3 mice). Almost all of the RFP-labeled presumptive stem cells showed the α and β morphologies we described in the previous work (61.5 ± 11.6 cells per mm^2^, *n* = 3 ventricular walls). The RFP+ cells with the α and β morphologies were highly branched at the cell body in contrast to the B1 type stem cells and are referred to in this work as the *Lrig1*-lineage stem cells. Almost all of the *Lrig1*-lineage stem cells were EdU- (Fig. 1C, 94.2 ± 3.6%, *n* = 3 ventricular walls) indicating that these cells had not entered S-phase during the time of tamoxifen-induced RFP labeling.

Then, we analyzed the S100 and RFP immunoreactivities in the *Lrig1* reporter mouse line as we analyzed the Tg(Nr2e1-EGFP) mouse line. In contrast to the *Nr2e1* reporter mouse line, we did not observe many ventricle-contacting apical extensions between the S100+ cells from the *Lrig1*-lineage stem cells (Fig. 1C). Nevertheless, consistent with the locations of the EGFP+ KI-67- ASCL1- cells in the Tg(Nr2e1-EGFP) mice, the cell bodies of the *Lrig1*-lineage stem cells were also in the subventricular zone under the S100+ ependymal cells (Fig. 1C).

We then analyzed additional neural stem cell reporter mouse lines to determine whether the observation above holds true. The Coffey *Lrig1*^*creERT2*^ knock-in knock-out mouse line [35] and *Pdgfrb*^*P2A-creERT2*^ knock-in mouse line [36, 37] were analyzed using the same method (Fig. 1D). When the locations of the RFP+ cells with the stem cell morphologies, i.e., branches or no branches, round cell body, and a long basal process, were quantitated, similar distributions were obtained from different reporter mice, without statistically significant differences (Fig. 1E, Student’s t test; Fig. 1F, Kolmogorov-Smirnov test). Therefore, with or without the apical extension that contacts the ventricle, the adult stem cells in the adult mouse brain ventricular-subventricular zone have their cell bodies located in the subventricular zone under the S100+ ependymal cells. Finally, the *Pdgfrb* reporter labeled more ventricle-contacting cells than the *Lrig1* reporters (Fig. 1G, Chi-square test, *p* < 0.01).

### The *Lrig1*-lineage stem cells in the adult brain largely do not proliferate during juvenile development

Above, we molecularly defined a population of quiescent neurogenic stem cells in the subventricular zone of the lateral wall. We then examined whether these *Lrig1*-lineage stem cells had proliferated earlier during juvenile development using thymidine analog labeling. Control experiments were performed first.

**Fig. 2:**
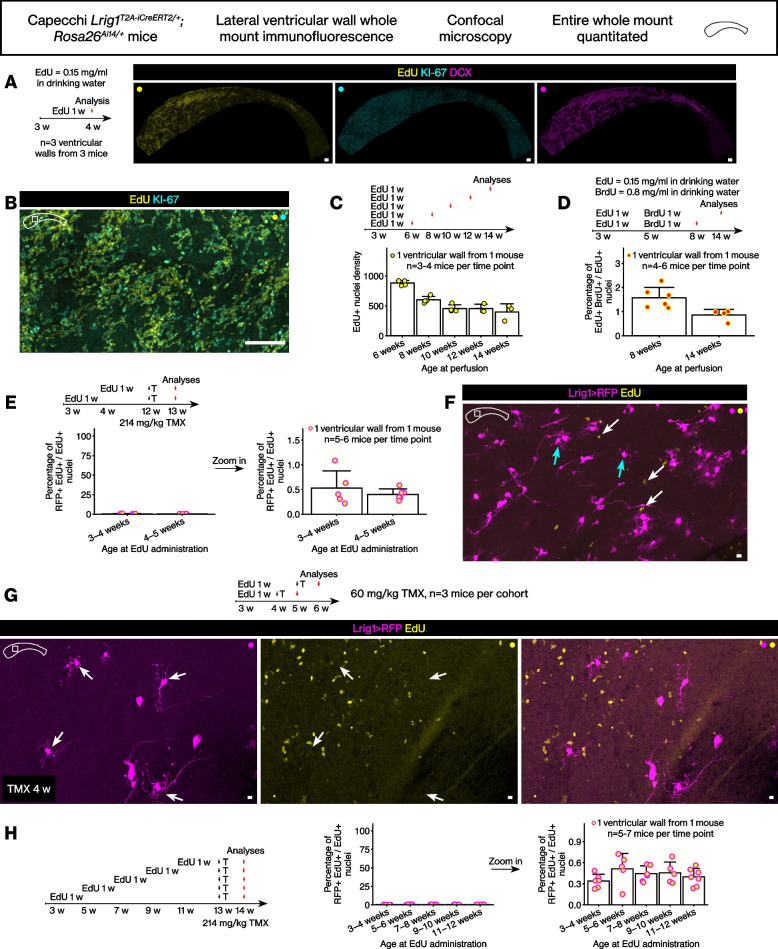
The *Lrig1*-lineage stem cells in the adult brain lateral wall were largely quiescent during juvenile development. **A** Control analysis of EdU administration in juvenile mice showed complete labeling without disrupting the KI-67+ cell or DCX+ cell numbers. Scale bar, 100 μm. **B** The EdU signal overlapped completely with the KI-67 signal. Scale bar, 100 μm. **C** EdU pulse-chase during juvenile development. Number of EdU+ nuclei per mm^2^. Mean ± standard deviation. **D** EdU then BrdU double pulse-chase during juvenile development. Mean ± standard deviation. **E** EdU pulse-chase during juvenile development then tamoxifen induction in adult age. Mean ± standard deviation. **F** A representative confocal image from one of the ventricular walls quantitated. Scale bar, 10 μm. **G** EdU pulse-chase during juvenile development then tamoxifen induction shortly after. A representative confocal image. Scale bar, 10 μm. **H** Additional time points of EdU pulses during juvenile development then tamoxifen induction in adults. Mean ± standard deviation

Previously, an EdU dose titration was performed with 12 week old mice [26], and we tested the same low dose of EdU on juvenile mice (postnatal day 21 to postnatal day 28). The administration of the EdU at 0.15 mg/ml in drinking water did not disrupt expression patterns of proliferation marker KI-67 and neuroblast marker DCX in the lateral wall (Fig. 2A-B, *n* = 3 ventricular walls from 3 mice). Next, we quantitated how much of the EdU labeling is diluted out by the continuous proliferation in the lateral wall. Mice were administered EdU for 1 week, then chased up to 14 weeks of age. Although the density of the EdU+ cells (the number of EdU+ nuclei / mm^2^) decreased by approximately half by 14 weeks, a significant number of EdU+ cells remained (Fig. 2C, *n* = 3–4 ventricular walls from 3 to 4 mice per time point). Because EdU toxicity can reduce proliferation, we then examined whether the EdU+ cells are in fact continuing to divide using a second pulse of BrdU. After sequential 1 week pulses of EdU then BrdU, mice were examined at 8 and 14 weeks of age. Decreasing percentage of EdU+ BrdU+ / EdU+ nuclei from 8 to 14 weeks indicated that the EdU+ cells were in fact continuing to divide, suggesting minimized toxicity from the low dose EdU pulse (Fig. 2D, *n* = 4–6 ventricular walls from 4 to 6 mice).

The *Lrig1*^*T2A-iCreERT2/+*^*; Rosa26*^*Ai14/+*^ mice were pulsed with EdU for 1 week starting at 3 and 4 weeks of age, then injected with tamoxifen at 12 weeks of age to label the *Lrig1*-lineage stem cells. The lateral walls from these mice were analyzed as above. This analysis revealed very low percentage of RFP+ EdU+ cells / EdU+ cells, indicating that more than 99% of the previously proliferated EdU+ cells were not labeled by the *Lrig1* reporter allele (Fig. 2E-F, *n* = 5–6 ventricular walls from 5 to 6 mice).

To determine whether an earlier tamoxifen induction reveals greater percentage of RFP+ EdU+ double-positive cells, the *Lrig1*^*T2A-iCreERT2/+*^*; Rosa26*^*Ai14/+*^ mice were administered EdU from 3 to 4 weeks, then tamoxifen was injected at 4 and 5 weeks of age to label the *Lrig1*-expressing cells. Analysis of the lateral walls from these mice after 1 week indicated that even with an earlier tamoxifen induction, almost all EdU+ cells were still RFP- as before (Fig. 2G, 0 RFP+ EdU+ cells per mm^2^ / 613 ± 264 EdU+ cells per mm^2^, *n* = 3 mice per time point).

Finally, we repeated and extended the analysis above with additional time points of EdU administration. EdU was administered starting from 3 weeks of age to 11 weeks of age. Then, tamoxifen was injected at 13 weeks of age and mice perfused after 1 week. The percentage of RFP+ EdU+ cells / EdU+ cells was again less than 1% across the entire time points (Fig. 2H, *n* = 5–7 ventricular walls from 5 to 7 mice).

**Fig. 3:**
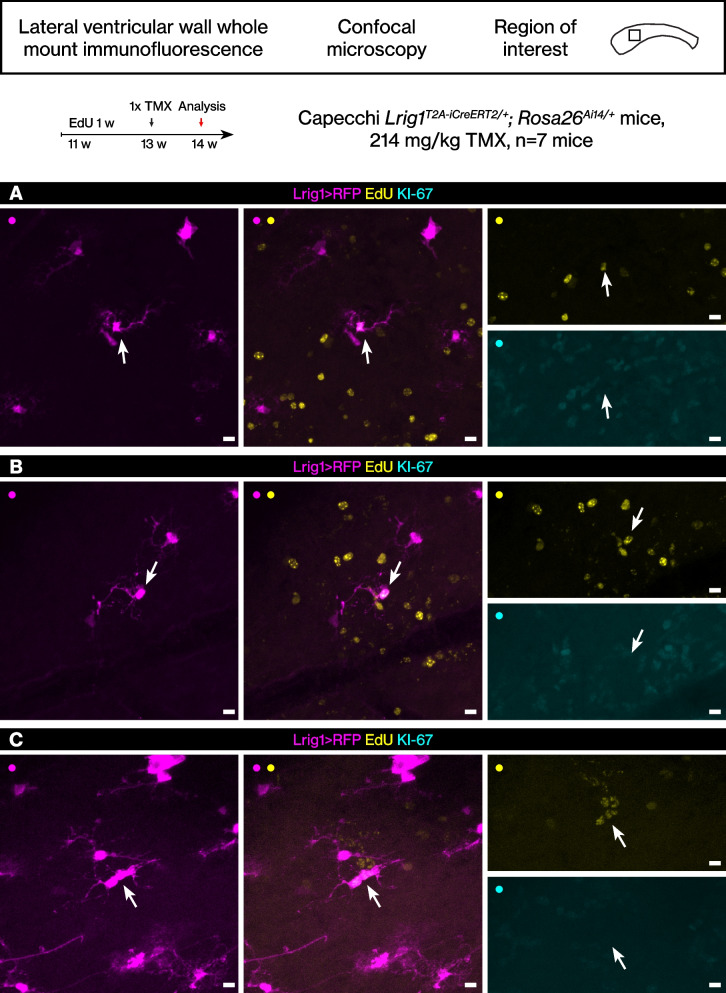
The rare EdU label-retaining *Lrig1*-expressing cells. **A**-**C** The rare RFP+ EdU+ cells were identified from low magnification confocal scans then imaged again with a high magnification objective at the confocal. Scale bar, 10 μm

Although only less than 1% of the EdU+ cells were RFP+ EdU+ double-positive cells, we identified the rare singlet RFP+ EdU+ double-positive cells to document their cellular phenotype. The RFP+ EdU+ cells were KI-67- (Fig. 3A-B), and therefore they were label-retaining cells, i.e., stem cells that had proliferated in the past but were not proliferating at the time of perfusion. These cells showed a long basal process without many branches at the cell body. Thus, the morphology of these RFP+ EdU+ cells clearly differed from the morphology of the *Lrig1*-lineage stem cells. Rare doublet cells were also identified (Fig. 3C).

### Morphologically distinct populations of stem cells can be labeled with the *Lrig1*^*T2A-iCreERT2*^ allele at juvenile age

**Fig. 4:**
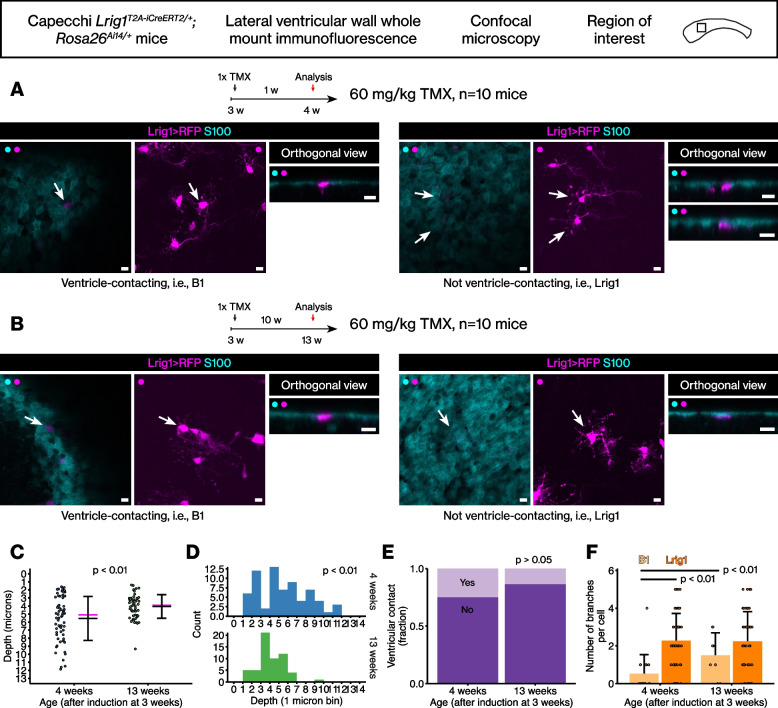
The *Lrig1*-expressing cells in the juvenile brain lateral wall. **A** Two morphologically distinct cells from tamoxifen induction during juvenile development. Scale bar, 10 μm. **B** The two distinct morphotypes remained after juvenile development. Scale bar, 10 μm. **C** Dot plot of the distributions of the cell body locations. Black bars, mean ± standard deviation. Magenta bar, median. Student’s t test. **D** Histogram of the same distribution. Kolmogorov-Smirnov test. **E** Percentages of ventricle-contacting or non-ventricle-contacting cells. Chi-square test. **F** Numbers of the cells’ branches during juvenile development. Mean ± standard deviation. Student’s t test

Because the EdU pulse-chase during juvenile development and tamoxifen induction in adults did not identify many RFP+ EdU+ double-positive cells, we analyzed the *Lrig1*-expressing cells from an earlier age to identify proliferating cells. Thus, the *Lrig1*^*T2A-iCreERT2/+*^*; Rosa26*^*Ai14/+*^ mice were induced with tamoxifen at postnatal day 21 (3 weeks of age). The analysis of the *Lrig1*^*T2A-iCreERT2/+*^*; Rosa26*^*Ai14/+*^ mice during juvenile development revealed at least 2 morphologically distinct populations. When tamoxifen induced at 3 weeks and analyzed at 4 weeks, we could easily identify many RFP-labeled cells that fit the description of the canonical B1 type stem cells, i.e., the cells with a round cell body, few branches, a long basal process, and an apical extension that contact the ventricle (Fig. 4A, *n* = 10 ventricular walls from 10 mice). In addition to the B1 type stem cells, we also identified RFP-labeled cells that were similar to the *Lrig1*-lineage stem cells that we observed in the adult brain, i.e., the cells with many branches at the cell body, no ventricular contact, and a long basal process (Fig. 4A).

We then analyzed the mice induced during postnatal day 21 at 13 weeks of age. At this age, we could still observe the two morphotypes of the RFP+ stem cells, i.e., the first one that contacts the ventricle as well as the second one that does not and is branched (Fig. 4B, *n* = 10 ventricular walls from 10 mice).

The locations of the two morphotypes of the RFP+ stem cells – i.e., the B1 type stem cells and the *Lrig1*-lineage stem cells – were quantitated from 3 weeks of age to 4 and 13 weeks of age (Fig. 4C, Student’s t test, *p* < 0.01). The change in the distribution of cells’ locations over time suggested divergent fates for the cells (Fig. 4D, Kolmogorov-Smirnov test, *p* < 0.01, Fig. 4E, Chi-square test, *p* > 0.05). Next, the numbers of branches in the RFP+ stem cells were quantitated (Fig. 4F). For the *Lrig1*-lineage stem cells, the number of branches did not increase significantly during juvenile development (Student’s t test, *p* > 0.05), suggesting that these cells were already in place by 4 weeks of age. However, for the ventricle-contacting B1 type stem cells, (1) they showed fewer branches than the *Lrig1*-lineage stem cells at 4 weeks of age (Student’s t test, *p* < 0.01) and (2) the number of the branches increased from 4 to 13 weeks (Student’s t test, *p* < 0.01). In other words, at 13 weeks of age, even the ventricle-contacting B1 type stem cells showed more branches than at an earlier time point.

### Selective proliferation of a stem cell subset during juvenile development

**Fig. 5:**
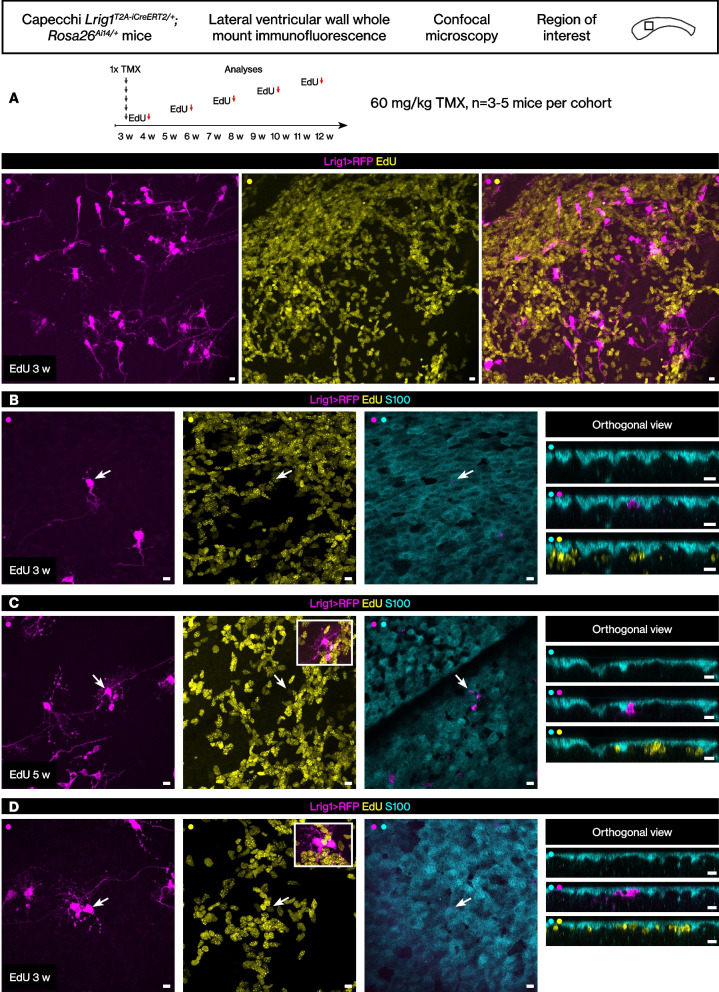
The *Lrig1*-expressing cells were largely quiescent during juvenile development. **A** Tamoxifen induction then EdU pulse during juvenile development. Two morphologically distinct subsets were again observed. Scale bar, 10 μm. **B** An RFP+ cell that was EdU-. Scale bar, 10 μm. **C** An RFP+ cell that was dimly EdU+. Scale bar, 10 μm. **D** A doublet of RFP+ EdU+ cells that did not contact the ventricle and were located in the subventricular zone. Scale bar, 10 μm

The analyses above suggested different behaviors of the two stem cell subsets in the ventricular-subventricular zone leading to divergent fates. We determined whether cellular proliferation is different among these subsets. Thus, after tamoxifen induction of the *Lrig1* reporter mice at 3 weeks of age, EdU was administered for 1 week until 13 weeks of age. The mice were perfused at the end of the EdU administration, such that all S-phase cells could be detected without the confounding EdU label dilution over time. Again, the B1 type stem cells as well as the *Lrig1*-lineage stem cells were revealed (Fig. 5A, *n* = 3–5 ventricular walls from 3 to 5 mice per time point). Surprisingly, most of the RFP+ stem cells of both subsets still had not gone through S-phase from 3 to 12 weeks of age (week 3 time point quantitation, 42.2 ± 7.6 RFP+ cells per mm^2^, 1.0 ± 0.91 RFP+ EdU+ cells per mm^2^, 2.4 ± 2.3% RFP+ EdU+ cells / RFP+ cells).

Thus, we searched for the rare singlet RFP+ stem cells that were also EdU+. The very rare singlet RFP+ EdU+ double-positive stem cells that were found and imaged at higher magnification were all B1 type stem cells (6 cells from the 10 mice of the first two time points). The cells that initially contacted the ventricle with an apical extension were EdU- (Fig. 5B). They entered the cell cycle to incorporate the EdU into their genomic DNA (Fig. 5C). Subsequently, these cells completed mitosis to form doublets (Fig. 5D). Intriguingly, we did not find any singlet RFP+ EdU+ double-positive stem cells that did not contact the ventricle and were branched at the cell body.

### Origin of the distinct stem cell subsets in the postnatal brain

**Fig. 6:**
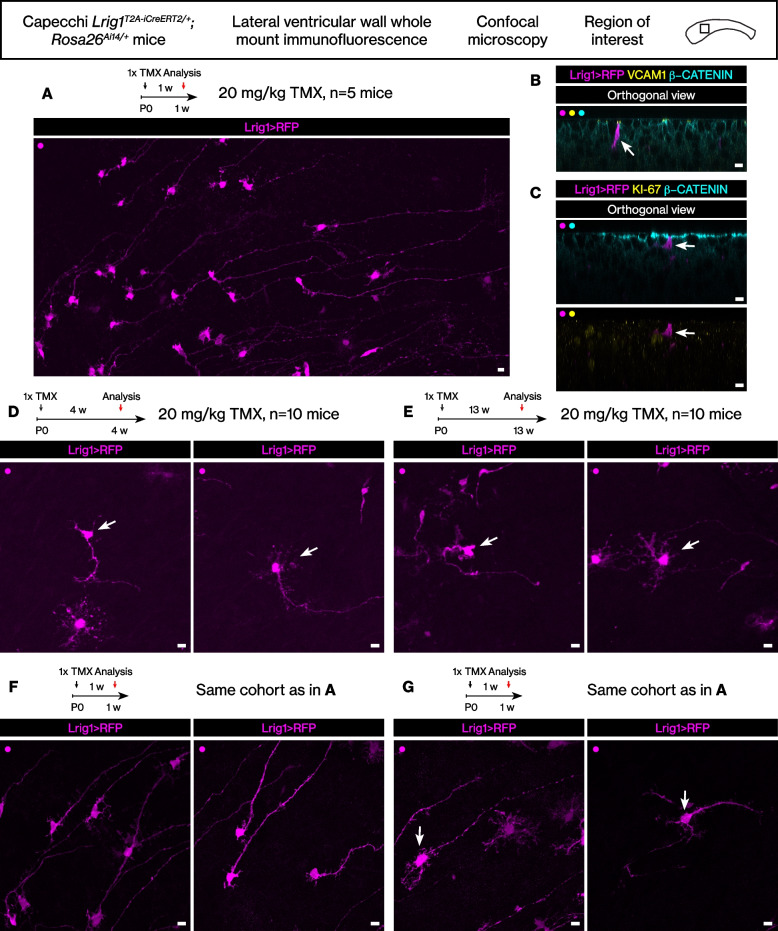
The *Lrig1*-expressing cells in the postnatal brain lateral wall. **A** RFP+ postnatal radial glial cells from tamoxifen induction during postnatal development. Scale bar, 10 μm. **B** VCAM1 expression in an RFP+ cell. Scale bar, 10 μm. **C** An RFP+ KI-67- cell. Scale bar, 10 μm. **D** Two distinct morphotypes at juvenile age after postnatal tamoxifen induction. Scale bar, 10 μm. **E** Two distinct morphotypes at young adult age after postnatal tamoxifen induction. Scale bar, 10 μm. **F** Unbranched RFP+ postnatal radial glial cells. Scale bar, 10 μm. **G** Branched RFP+ postnatal radial glial cells. Scale bar, 10 μm

Because the distinct morphotypes of the largely quiescent stem cells were already in place at 4 weeks of age, we determined whether they originate from the same pool of progenitor cells earlier on, or whether there exist entirely different progenitor pools for the different morphotypes of adult stem cells. We injected tamoxifen to newborn *Lrig1*^*T2A-iCreERT2/+*^*; Rosa26*^*Ai14/+*^ pups at postnatal day 0 then analyzed the mice at 1 week, 4 weeks, or 13 weeks of age. When analyzed at 1 week, we observed many RFP+ postnatal radial glial cells [24] that showed a round cell body and a very long basal process (Fig. 6A, *n* = 5 ventricular walls from 5 mice). The RFP+ postnatal radial glial cells were all VCAM1+ [38] and mostly KI-67- (RFP+ KI-67- cells, 92.3 ± 3.0% of all RFP+ postnatal radial glial cells, 120.1 ± 44.2 cells per mm^2^), suggesting they were quiescent at the time of perfusion (Fig. 6B). Importantly, we did not observe cytoarchitecturally distinct subsets of the RFP+ postnatal radial glial cells – almost all of the RFP+ postnatal radial glial cells had cell body under the ventricular wall and contacted the ventricle with an apical extension (Fig. 6C).

The analyses of the *Lrig1* reporter mice at later time points showed the RFP+ stem cells of both morphotypes (Fig. 6D-E, *n* = 10 ventricular walls from 10 mice per time point). Thus, both subsets of the stem cells originated from a single pool of postnatal radial glial cells. Commencing between postnatal day 0 and 4 weeks of age, the single pool bifurcated into two pools, one ventricle-contacting and less branched and one non-ventricle-contacting and highly branched. Indeed, we observed that some RFP+ postnatal radial glial cells at 1 week of age already showed more branches than the rest (Fig. 6F-G) consistent with the notion that the branched postnatal radial glial cells will later become the branched *Lrig1*-lineage stem cells.

### Fate of the stem cell subset that proliferates during postnatal/juvenile development

**Fig. 7:**
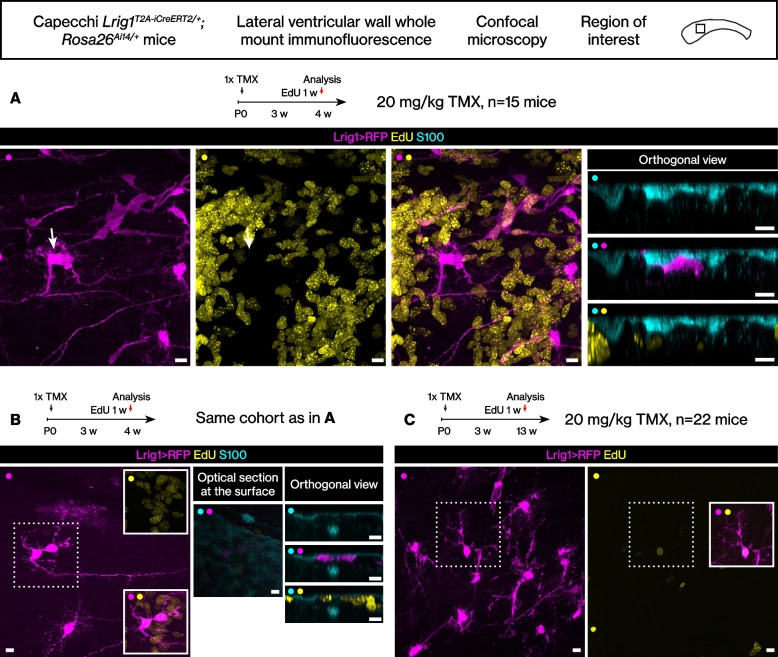
Fate of the *Lrig1*-expressing cells during postnatal/juvenile development. **A** Tamoxifen induction postnatally and EdU pulse during juvenile development. A doublet of RFP+ EdU- cells located in the subventricular zone. Scale bar, 10 μm. **B** A doublet of RFP+ EdU+ cells located in the subventricular zone. Scale bar, 10 μm. **C** Tamoxifen induction postnatally and EdU pulse-chase from juvenile development to young adult age. A singlet RFP+ EdU+ label-retaining cell. Scale bar, 10 μm

We further analyzed the proliferative behaviors of the distinct subsets. The *Lrig1*^*T2A-iCreERT2/+*^*; Rosa26*^*Ai14/+*^ mice were tamoxifen induced at postnatal day 0, administered EdU at 3 weeks of age for 1 week, then they were perfused at 4 weeks or 13 weeks of age. When analyzed at 4 weeks, as above, the RFP+ EdU+ stem cells were again rare (6.7 ± 2.6 cells per mm^2^, 6.3 ± 1.7% RFP+ EdU+ cells / RFP+ cells, *n* = 15 ventricular walls from 15 mice). However, consistent with a proliferative history during postnatal/juvenile development, we observed doublets of RFP+ stem cells that were EdU- (Fig. 7A) or EdU+ (Fig. 7B). Next, when analyzed at 13 weeks, we observed extremely rare singlet RFP+ EdU+ double-positive cells (Fig. 7C, 3 cells from *n* = 22 ventricular walls from 22 mice). The morphology of the singlet RFP+ EdU+ cells differed from the stem cells already described above. They were not branched at the cell body and showed a more complex basal process.

### *Lrig1* knock-out results in hyperproliferation without increased apoptosis

Taken together, the lineage tracing analyses above suggested that *Lrig1* is expressed in all known subsets of quiescent stem cells through postnatal and juvenile development. We then analyzed the lateral walls of *Lrig1* knock-out mice for cellular phenotypes to assess its function. Two mouse lines with different *Lrig1* null alleles were utilized. First was the Coffey *Lrig1*^*creERT2*^ allele [35] that replaces the first exon sequence encoding the membrane-localizing signal sequence with the creER^T2^ cDNA. Second was the EUCOMM *Lrig1*^*Δ*^ allele that results in a non-sense frame-shift prior to the protein’s transmembrane domain because of cre recombination of a floxed *Lrig1* allele [39]. The Coffey knock-out and wildtype littermate mice were analyzed at 1 year and 3 months and at 2 years and 4 months. The EUCOMM knock-out and wildtype littermate mice were analyzed at 1 year and 3 months. To assess any confounding effect of the genetic background, the Coffey knock-out and wildtype littermate mice from a later generation of backcross to the C57BL/6J background were analyzed at 1 year and 8 months.

**Fig. 8:**
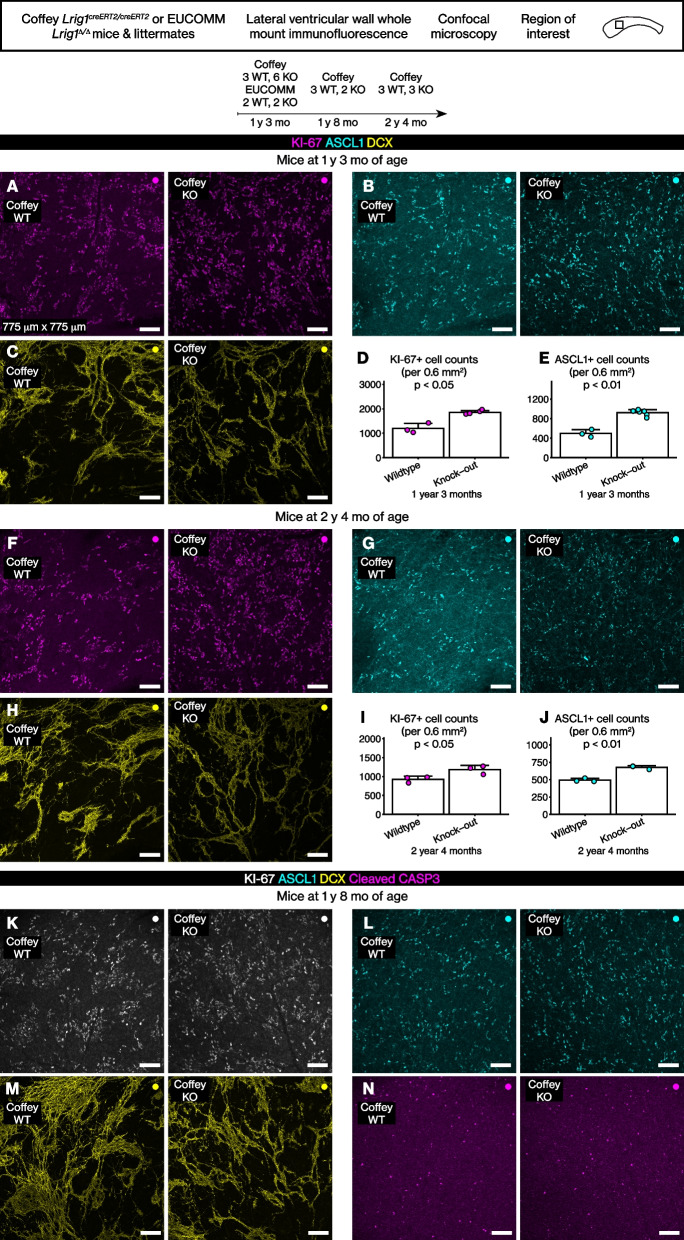
*Lrig1* knock-out resulted in persistent hyperproliferation in the lateral wall even in old mice. **A**-**C** KI-67, ASCL1, or DCX immunoreactivity in 1 year and 3 month-old mice. Scale bar, 100 μm. **D**-**E** Graphs of KI-67+ and ASCL1+ cell counts. Mean ± standard deviation. Student’s t test. **F-H** KI-67, ASCL1, or DCX immunoreactivity in 2 years and 4 month-old mice. Scale bar, 100 μm. **I-J** Graphs of KI-67+ and ASCL1+ cell counts. Mean ± standard deviation. Student’s t test. **K-N** KI-67, ASCL1, DCX, or cleaved CASP3 immunoreactivity in 1 year and 8 month-old mice. Scale bar, 100 μm

Remarkably, we observed increased numbers of KI-67+ cells and ASCL1+ cells in every knock-out mouse at every age (Fig. 8A-J, Student’s t test). Although the numbers of all DCX+ neuroblasts could not be counted directly because of the compacted DCX staining pattern, the numbers of early neuroblasts (i.e., the KI-67+ cells that are ASCL1- DCX+) were increased in the knock-out mice. The GFAP immunoreactivity was unchanged in the knock-out mice (Additional file 1 A-B). The same phenotype was observed in the Coffey and the EUCOMM knock-out mice (Fig. 8A-E, Additional file 2 A-C). The phenotype was not altered by the increasing contribution of the C57BL/6J genetic background (Fig. 8A-C, K-M).

Then, we analyzed whether apoptosis is increased because of the increased proliferation in the lateral wall stem cell niche. Analysis of cleaved CASP3 revealed no difference between the Coffey and EUCOMM knock-out and wildtype littermate mice (Fig. 8N, Additional file 2 D, Student’s t test, *p* > 0.05). Finally, we did not observe any tumors in the brains of these mice.

## Discussion

In this work, we analyzed the development of the *Lrig1*-lineage stem cells in the lateral wall during postnatal and juvenile ages. We also determined the long-term effect(s) of knocking out *Lrig1* on cellular proliferation in the lateral wall.

Our results are consistent with a view that there are at least two distinct subsets of adult stem cells in the lateral wall of the adult mouse brain. There are the cells we termed the *Lrig1*-lineage stem cells with the α and β morphologies [21]. These cells resembled the previously identified branched B type stem cells [19]. These cells could be labeled preferentially when the *Lrig1*^*T2A-iCreERT2/+*^*; Rosa26*^*Ai14/+*^ mice were induced during adult age (12–13 weeks). In addition, there are the previously identified B1 type stem cells [20]. These cells could be revealed with the Tg(Nr2e1-EGFP) transgenic mice, with the *Pdgfrb* knock-in mice, and when the *Lrig1*^*T2A-iCreERT2/+*^*; Rosa26*^*Ai14/+*^ mice were induced during postnatal age (postnatal day 0) or juvenile age (postnatal day 21).

Currently, it is not clear why the *Lrig1*-lineage labeling from the adult induction or the postnatal/juvenile induction differ. It may be that certain subsets of the stem cells are more abundant at different ages and/or that there is a molecular difference between the subsets at different ages, including a difference in the *Lrig1* expression levels.

Regardless, our analyses suggested the bifurcation of the two subsets from a common progenitor pool, the postnatal radial glial cells [24]. The two subsets seemed to be established as adult stem cells by 4 weeks of age. Around that age, we also observed the selective proliferation of the B1 type stem cell subset. We presume this generated the B2 type stem cells and leads to the more rapid depletion of the B1 type stem cells during aging [40, 41].

The initial observation by Obernier et al. implies that the cells we termed the *Lrig1*-lineage stem cells, i.e., the branched B type stem cells [19], are less likely to be activated during postnatal/juvenile development and are more likely to remain quiescent until adult age. It then follows that these cells would be the long-term adult stem cells in the adult brain. This assertion is supported by our previous observations of RFP-labeled neuroblasts and interneurons from this lineage throughout adult life, as late as 2 years and 6 months of age [21–23].

Our previous work demonstrated a low level of the LRIG1 protein in the lateral wall cells from the adult mouse brain [21]. From the highly penetrant *Lrig1* knock-out phenotype in the lateral wall we described here, we infer that this low level of the LRIG1 protein expression is nevertheless indispensable even in the presence of other *Lrig* genes, *Lrig2* and *Lrig3*.

Our observation that the proliferation in the lateral wall did not exhaust in the old *Lrig1* knock-out mice – although perhaps not surprising given previous observations [35, 42] – is nevertheless notable. It is reported for many years that compromised stem cell quiescence leads to stem cell depletion over time (for example, [43]). As we and others have documented [21, 42, 44], *Lrig1* is expressed in neural progenitors during embryonic and postnatal development and in adult stem cells later on. We demonstrated here that these *Lrig1*-lineage labeled adult stem cells that are active later in the adult brain were largely not proliferating during postnatal/juvenile development, consistent with the notion that *Lrig1* maintains their quiescence. Thus, the unexpected persistence of the hyperproliferation in the old *Lrig1* knock-out mice suggests the functions of the LRIG1 protein may not be limited to the maintenance of quiescence in the adult stem cells. Indeed, *Lrig1* may act on the adult stem cell pool at multiple stages of development to limit cellular proliferation in the adult brain. Thus, conditional mutagenesis studies in the future may elucidate the multiple functions of the LRIG1 protein over time.

The future studies may also reveal how *Lrig1* constrains the adult stem cell pool size and whether it is possible to exploit that knowledge to increase the number of the long-term stem cells in the adult brain. Achieving that may be possible via inducing the proliferation of the quiescent stem cells during postnatal/juvenile development. Or, perhaps their specification and allocation during embryogenesis [2–4], a process in which *Lrig1* was already implicated [44], should be further investigated.

## Conclusion

*Lrig1* is expressed in quiescent stem cells of postnatal, juvenile, and adult mouse brain. It is likely involved in multiple aspects of stem cell pool size regulation including the maintenance of quiescence.

## Supplementary Information

**Additional file 1. **GFAP immunoreactivity in the Coffey *Lrig1* mice. A. GFAP immunoreactivity in 1 year and 3 month-old mice. Scale bar, 100 microns. B. GFAP immunoreactivity in 2 years and 4 month-old mice. Scale bar, 100 microns.

**Additional file 2. ***Lrig1* knock-out phenotype in the EUCOMM *Lrig1*^*Δ/ Δ*^ mice. A-D. KI-67, ASCL1, DCX, or cleaved CASP3 immunoreactivity in 1 year and 3 month-old mice. Scale bar, 100 microns.

## Data Availability

The plasmids generated during the course of this work were deposited at Addgene. The mouse lines generated were deposited at The Jackson Laboratory and the MMRRC. Data will be deposited at Figshare.

## Abbreviations

Ascl1: Achaete-scute family bHLH transcription factor 1
BAC: Bacterial artificial chromosome
BrdU: Bromo deoxyuridine
BSA: Bovine serum albumin
Casp3: Caspase 3
Dcx: Doublecortin
EdU: Ethynyl deoxyuridine
EGFP: Enhanced green fluorescent protein
EUCOMM: European conditional mouse mutagenesis
Gfap: Glial fibrillary acidic protein
GFP: Green fluorescent protein
IgG: Immunoglobulin gamma
Lrig1: Leucine-rich repeats and immunoglobulin-like domains 1
Nr2e1: Nuclear receptor subfamily 2, group E, member 1
PBS: Phosphate buffered saline
PBST: Phosphate buffered saline plus Triton X-100
Pdgfrb: Platelet derived growth factor receptor, beta polypeptide
PLP fixative: Periodate-lysine-paraformaldehyde fixative
RFP: Red fluorescent protein
RNA: Ribonucleic acid
Rosa26: Gt(ROSA)26Sor, gene trap ROSA 26, Philippe Soriano
TBST: Tris buffered saline plus Triton X-100
Tlx: Tailless
Vcam1: Vascular cell adhesion molecule 1
