# Correction: *Lrig1* expression identifies quiescent stem cells in the ventricular‑subventricular zone from postnatal development to adulthood and limits their persistent hyperproliferation

**DOI:** 10.1186/s13064-023-00172-0

**Published:** 2023-05-23

**Authors:** Hyung-song Nam, Mario R. Capecchi

**Affiliations:** Department of Human Genetics, University of Utah School of Medicine, Salt Lake City, UT 84112‑5331 USA

**Correction: Neural Dev 18, 1 (2023)**

10.1186/s13064-022-00169-1

The authors would like to correct errors and update two figures in the original publication of the article [1].

1. Page 2, “All mouse lines were backcrossed for at least 6 generations” corrected to “All mouse lines were backcrossed for at least 4 generations”.

2. Page 4, “0.1 M boric acid pH 8” corrected to “0.1 M sodium borate pH 8”.

3. Page 4, after “RFP goat polyclonal Rockland Immunochemicals 200-101-379 1:500” and before “RFP rabbit polyclonal Rockland Immunochemicals 600-401-379 1:500”, addition of “RFP guinea pig polyclonal Frontier Institute MSFR105900 1:500”.

4. Page 4, “To our knowledge, this mouse line was not previously characterized.” corrected to “To our knowledge, this mouse line was not previously characterized in detail.”.

5. Page 6, “KI-67 + ASCL1 + EGFP + cells, 3373 ± 532 cells per mm^2^” corrected to “EGFP + KI-67 + ASCL1 + cells, 3373 ± 532 cells per mm^2^”.

6. Page 6, “Consistent with the notion that the EGFP + KI-67- ASCL1- cells are quiescent B1 type stem cells” corrected to “Consistent with the notion that at least some of the EGFP + KI-67- ASCL1- cells are quiescent B1 type stem cells”.

7. Page 12, “all of the RFP + postnatal radial glial cells had cell body under the ventricular wall and contacted the ventricle with an apical extension (Fig. 6C).” corrected to “almost all of the RFP + postnatal radial glial cells had cell body under the ventricular wall and contacted the ventricle with an apical extension (Fig. 6C).”

8. Figure 3 was updated.

**Fig. 3:**
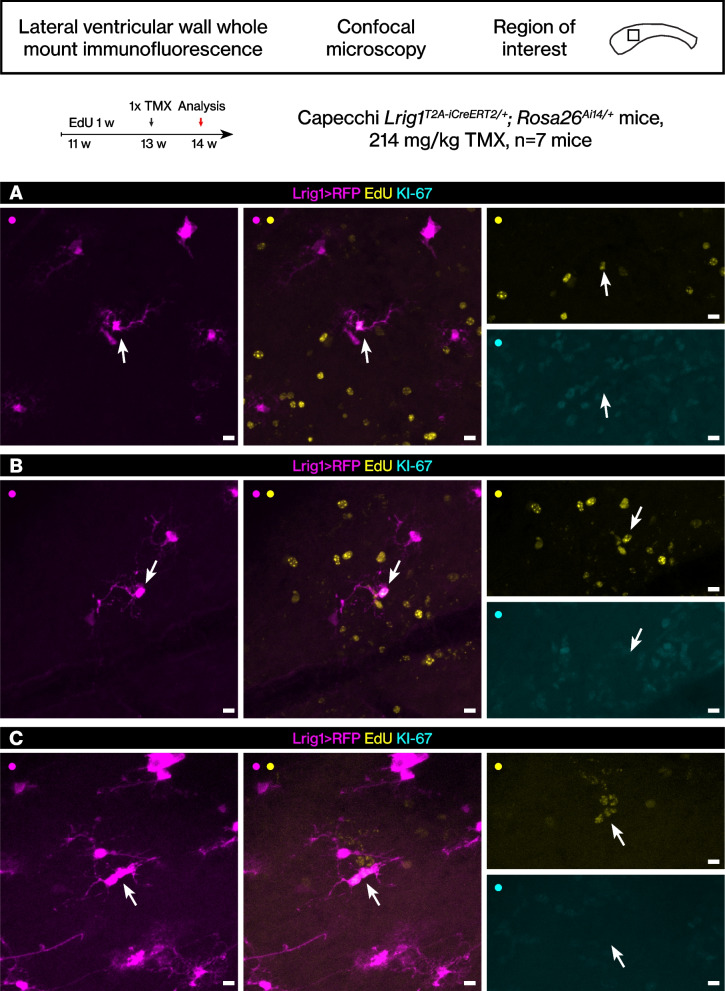
The rare EdU label-retaining Lrig1-expressing cells. **A**-**C** The rare RFP+ EdU+ cells were identified from low magnification confocal scans then imaged again with a high magnification objective at the confocal. Scale bar, 10 μm

9. Figure 6 was updated.

**Fig. 6:**
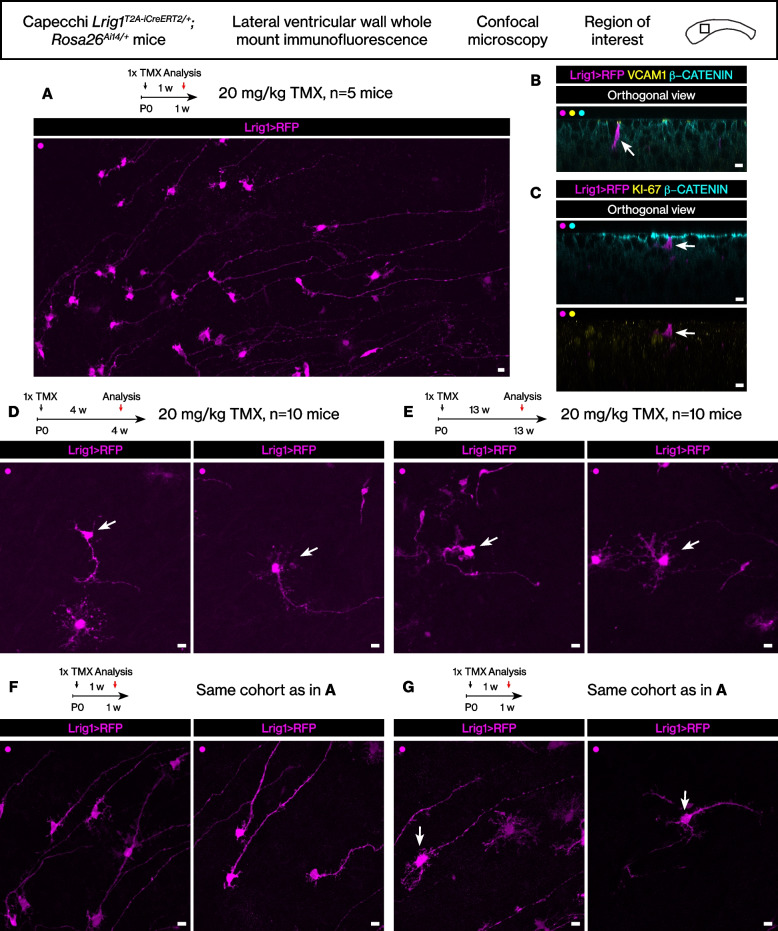
The Lrig1-expressing cells in the postnatal brain lateral wall. **A** RFP+ postnatal radial glial cells from tamoxifen induction during postnatal development. Scale bar, 10 μm. **B** VCAM1 expression in an RFP+ cell. Scale bar, 10 μm. **C** An RFP+ KI-67- cell. Scale bar, 10 μm. **D** Two distinct morphotypes at juvenile age after postnatal tamoxifen induction. Scale bar, 10 μm. **E** Two distinct morphotypes at young adult age after postnatal tamoxifen induction. Scale bar, 10 μm. F Unbranched RFP+ postnatal radial glial cells. Scale bar, 10 μm. G Branched RFP+ postnatal radial glial cells. Scale bar, 10 μm
