# Supplementary figures and images for: Lrig1 expression identifies quiescent stem cells in the ventricular-subventricular zone from postnatal development to adulthood and limits their persistent hyperproliferation
Source: Neural Dev. 2023 Jan 11;18:1. doi: 10.1186/s13064-022-00169-1 (PMC9832784; doi:10.1186/s13064-022-00169-1)

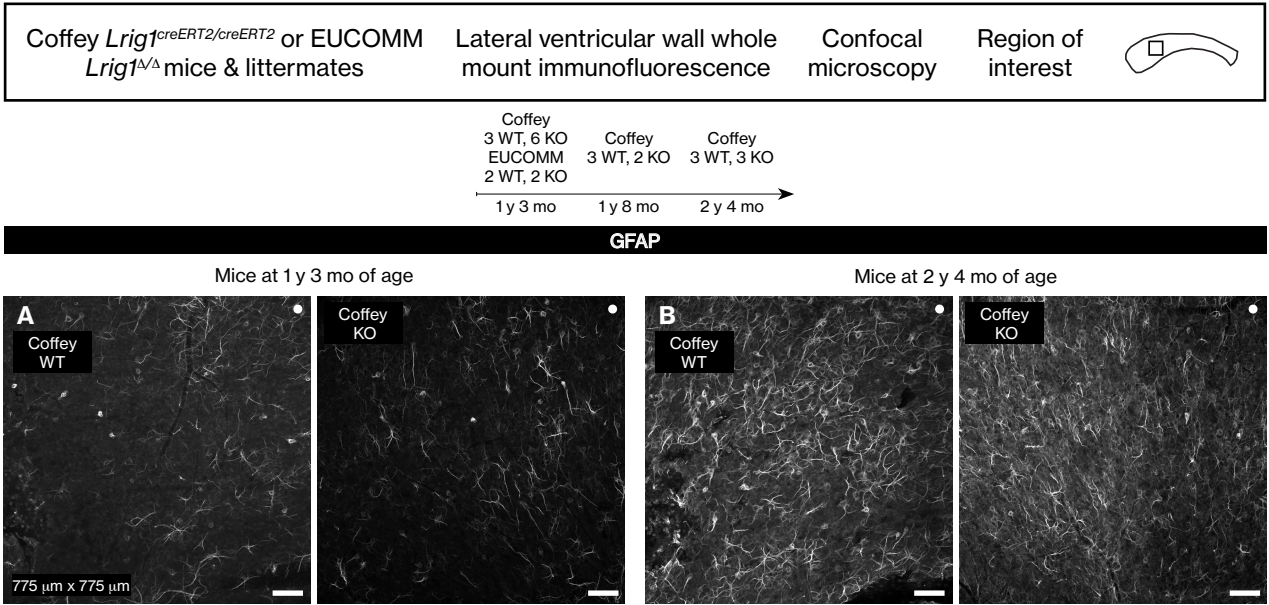

Supplement: Supplementary file 1 — Additional file 1. GFAP immunoreactivity in the Coffey Lrig1 mice. A. GFAP immunoreactivity in 1 year and 3 month-old mice. Scale bar, 100 microns. B. GFAP immunoreactivity in 2 years and 4 month-old mice. Scale bar, 100 microns. [file 13064_2022_169_MOESM1_ESM.pdf]

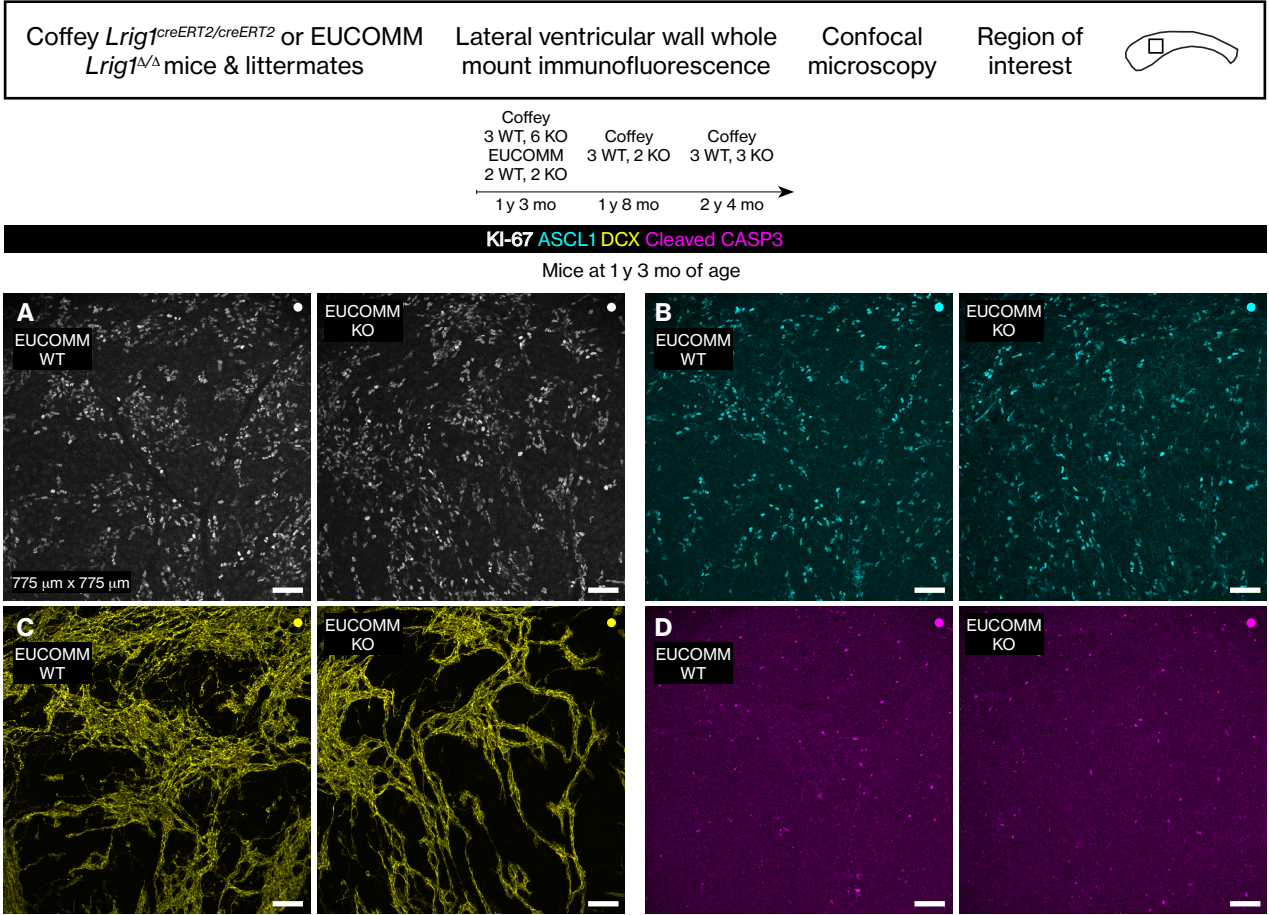

Supplement: Supplementary file 2 — Additional file 2. Lrig1 knock-out phenotype in the EUCOMM Lrig1Δ/ Δ mice. A-D. KI-67, ASCL1, DCX, or cleaved CASP3 immunoreactivity in 1 year and 3 month-old mice. Scale bar, 100 microns. [file 13064_2022_169_MOESM2_ESM.pdf]
